# Bioengineered nasal septum implant with 3D-printed silicone and chondrocyte-seeded fibrin hydrogel

**DOI:** 10.1093/rb/rbag122

**Published:** 2026-06-09

**Authors:** Emeline Perrier-Groult, Delphine Vertu-Ciolino, Fanny Brunard, Audrey Ziverec, Edwin Courtial, Marielle Pasdeloup, Maxime Ducret, Jérôme Lafont, Maria-Christina Saade, Christophe Marquette, Frédéric Mallein-Gerin, Jean-Daniel Malcor

**Affiliations:** Laboratory of Tissue Biology and Therapeutic Engineering, CNRS UMR 5305, University Claude Bernard and University of Lyon, 69367 Lyon Cedex 07, France; Institute for Regenerative Medicine and Biotherapy, INSERM UMR 1183, CHU Hôpital Saint Eloi, Montpellier, 34295, France; Laboratory of Tissue Biology and Therapeutic Engineering, CNRS UMR 5305, University Claude Bernard and University of Lyon, 69367 Lyon Cedex 07, France; Hôpital Edouard Herriot, Lyon, 69003, France; Laboratory of Tissue Biology and Therapeutic Engineering, CNRS UMR 5305, University Claude Bernard and University of Lyon, 69367 Lyon Cedex 07, France; Laboratory of Tissue Biology and Therapeutic Engineering, CNRS UMR 5305, University Claude Bernard and University of Lyon, 69367 Lyon Cedex 07, France; 3d.FAB, Univ Lyon, Université Lyon1, CNRS, INSA, CPE-Lyon, ICBMS, UMR 5246, 69622 Villeurbanne Cedex, France; Laboratory of Tissue Biology and Therapeutic Engineering, CNRS UMR 5305, University Claude Bernard and University of Lyon, 69367 Lyon Cedex 07, France; Laboratory of Tissue Biology and Therapeutic Engineering, CNRS UMR 5305, University Claude Bernard and University of Lyon, 69367 Lyon Cedex 07, France; Laboratory of Tissue Biology and Therapeutic Engineering, CNRS UMR 5305, University Claude Bernard and University of Lyon, 69367 Lyon Cedex 07, France; Laboratory of Tissue Biology and Therapeutic Engineering, CNRS UMR 5305, University Claude Bernard and University of Lyon, 69367 Lyon Cedex 07, France; 3d.FAB, Univ Lyon, Université Lyon1, CNRS, INSA, CPE-Lyon, ICBMS, UMR 5246, 69622 Villeurbanne Cedex, France; Laboratory of Tissue Biology and Therapeutic Engineering, CNRS UMR 5305, University Claude Bernard and University of Lyon, 69367 Lyon Cedex 07, France; Laboratory of Tissue Biology and Therapeutic Engineering, CNRS UMR 5305, University Claude Bernard and University of Lyon, 69367 Lyon Cedex 07, France

**Keywords:** nasal septum reconstruction, 3D-printed silicone, fibrin hydrogel, nasal chondrocytes, cartilage engineering

## Abstract

Nasal septum reconstruction remains a significant challenge when native cartilage is deficient due to trauma, congenital defects or oncological resection. Although autologous cartilage grafts remain the gold standard, their use is limited by donor site morbidity and tissue availability. Synthetic implants, while readily available, present rejection, infection and extrusion issues, which complicate their use in nasal reconstruction. Here, we present a bioengineered alternative, that is a hybrid nasal septum implant consisting of a 3D-printed medical-grade silicone scaffold combined with fibrin hydrogels seeded with human nasal chondrocytes (HNCs). HNCs cultured within the fibrin-silicone constructs under chondrogenic conditions (with BMP-2, insulin and triiodothyronine) exhibited robust re-differentiation, evidenced by an upregulated expression of cartilage-specific genes (*COL2A1*, *COL9A1*, *ACAN*) and matrix deposition (type II collagen). Subcutaneous implantation in immunodeficient mice confirmed the *in vivo* stability of the constructs, with preservation of the cartilaginous matrix. A full-scale implant was also fabricated and matured *in vitro*, demonstrating extracellular matrix production. Surgical handling in a human cadaver showed optimal anatomical fit and implant resilience. Our findings demonstrate that the combination of 3D-printed silicone scaffolds with fibrin–HNC matrices constitutes hybrid nasal septum implants, advancing toward clinically translatable nasal reconstruction.

## Graphical Abstract

**Graphical Abstract:**
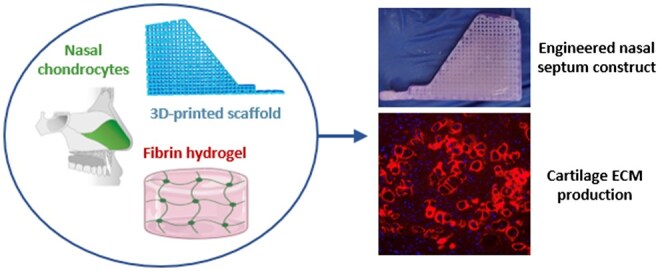

## Introduction

The nasal septum plays a pivotal role in both the breathing function of the nose and facial esthetics, contributing to airflow regulation, dorsal structural support and midline facial architecture. In cases of trauma, congenital anomalies, oncological resections or significant septal deviation, surgical reconstruction becomes essential, particularly when native cartilage is compromised or absent. Autologous cartilage grafts from septal, auricular or costal sources remain the current gold standard for septal reconstruction. However, these approaches are inherently limited by finite tissue availability that can cause donor site morbidity, and the difficulty in replicating the complex biomechanical and geometric properties of the native nasal septum [1–4].

Synthetic nasal implants offer an alternative to autologous grafting. Common materials include Gore-Tex (expanded PTFE), Medpor (porous polyethylene) and silicone-based compounds. These implants are readily available and avoid the need for tissue harvesting. However, their permanent presence in the body can lead to complications such as infection, uncontrolled immune response or extrusion [5]. To improve implant integration within the native tissue, such bioinert materials can be combined with cyto-compatible hydrogels or decellularized extracellular matrix (ECM) [6]. While Gore-Tex and Medpor are not amenable to standard additive manufacturing due to their physical and chemical properties, medical-grade silicone can be processed using advanced 3D printing techniques such as direct ink writing [7]. These techniques enable precise control over implant geometry and mechanical properties, allowing the creation of patient-specific structures [8]. Furthermore, 3D printing facilitates the creation of porous architectures that can be combined with biological hydrogels, thereby enhancing biocompatibility and actively promoting tissue integration. This approach enables the development of next-generation nasal implants that more closely replicate the characteristics of the native tissue and reduce the risk of postoperative complications.

Building on this premise, we developed a bioengineered nasal septum implant composed of a 3D-printed, medical-grade silicone scaffold seeded with a fibrin-based hydrogel containing expanded human nasal chondrocytes (HNCs). HNCs exhibit high proliferative and chondrogenic potential, making them excellent candidates for cartilage tissue engineering [9]. Fibrin, a natural biopolymer derived from the coagulation cascade, has been widely used as a hydrogel matrix for cartilage engineering due to its intrinsic cell-adhesive properties, biodegradability and ability to support chondrogenic differentiation [10]. In our approach, the constructs (composed of 3D-printed silicone scaffolds embedded in a chondrocyte-loaded fibrin gel) were matured *in vitro* using a chondrogenic medium supplemented with bone morphogenetic protein-2 (BMP-2), insulin and triiodothyronine (T3). This combination was originally identified as enhancing cartilage matrix production by human chondrocytes [11] and has been successfully applied to our tissue engineering protocols using articular or nasal chondrocytes in contact with various biomaterials [12–17].

To bridge the gap between *in vitro* development and clinical application, two types of 3D-printed silicone scaffolds were coated with fibrin encapsulated with HNCs: a square-shaped prototype, for subcutaneous implantation in the back of mice, to evaluate construct stability and biocompatibility; and a full-scale, anatomically-shaped septal implant to assess its handling and anatomical fit in a cadaveric surgical setting. Our gene and protein expression analyses, along with immunofluorescence data, confirmed chondrogenic differentiation and cartilage-like matrix production *in vitro*, with both the scaffold prototypes and the anatomically-shaped septal constructs. The scaffold prototypes demonstrated retention of their structural integrity and cartilaginous tissue formation *in vivo*. In parallel, the surgical placement of the full-size implant following native septum resection in a cadaver restored nasal morphology, demonstrating the potential for clinical translation. Taken together, our findings demonstrate the feasibility of engineering biologically active nasal septum implants and highlight their potential for future clinical applications in septal reconstruction.

## Materials and methods

### 3D printing of silicone implants

Silicone implants were 3D printed using medical-grade silicone (LSR4370, ELKEM Silicone, France) supplemented with 2% (w/w) medical-grade PEG400 (BASF France), as a yield stress agent [18]. Silicone 3D printing was carried out using a COSMED333 printer (TOBECA, France) equipped with a Vipro 3 head (VicoTec, Germany). STL files of the parts were sliced using Slic3r open-source software, using a 40% fill, a 200 µm printing nozzle and a 100 µm layer thickness. Implants were printed at 30 mm/s on a sterile Petri dish, incubated at room temperature (RT) for 14 h, followed by complete reticulation at 80°C for 12 h.

### 3D printing of molds 

The molds used to fabricate silicone-based scaffolds were 3D printed with an ObjetPro printer (Stratasys, USA) using VeroClear resin (Stratasys, USA). Post-processing involved removing the support material with a high-pressure water jet, followed by a 2-h UV post-cure step. Prior to cell-based experiments, 3D-printed molds and scaffolds were soaked in 70% EtOH for 5 min, dried and exposed to UV during 30 min for sterilization.

### Cell isolation and expansion

Samples of human nasal septum cartilage were collected from surgical waste from patients who had undergone septoplasty or rhinoseptoplasty at the Edouard Herriot Hospital in Lyon. They were collected with the written consent of the donors and complied with local ethical guidelines, national and European legislation on the collection and handling of human samples, and the protection of personal data (the protocol was approved by the French Ministry of Higher Education and Research’s ethics committee for research on human samples, CODECOH: DC-2014-2325). Experiments were conducted with HNCs isolated from 18 individual donors (donors labeled 1–18, see Table 1 for donor characteristics). The specific donors used in each experiment are indicated in the corresponding figure legends to ensure traceability. Donor-specific responses were analysed independently to account for biological variability.

**Table 1:** Age and sex of donors.

Donor	1	2	3	4	5	6	7	8	9	10	11	12	13	14	15	16	17	18
Gender	M	M	M	M	M	F	M	F	M	F	M	M	M	F	F	F	M	F
Age (years)	32	32	50	50	56	53	50	62	28	28	20	28	27	45	53	21	33	21

For HNC extraction, 0.2–0.5 g of septal cartilages were washed with phosphate-buffered saline (PBS) (Sigma-Aldrich) supplemented with 50 µg/mL streptomycin (Panpharma), then cut into small slices (2 mm^3^) and digested overnight at 37°C with 0.5 mg/mL bacterial collagenase A (Roche Applied Science) in a culture medium consisting of Dulbecco’s modified Eagle medium/HAM’s F12 (DMEM/F12) (Gibco/Life Technologies). The cell suspension was filtered and isolated chondrocytes were seeded at a density of 1.5 × 10^4^ cells on T75 culture flasks with culture media supplemented with 10% fetal bovine serum (FBS) (Gibco-Invitrogen), 50 µg/mL streptomycin, 5 ng/mL fibroblast growth factor-2 (FGF-2) (R&D Systems) and 5 μg/mL insulin (Umuline Rapide, Lilly). The combination of FGF-2 and insulin is referred to as FI. Complete medium supplemented with FI was replaced three times a week. HNCs were cultured for 2 weeks, then trypsinized.

### Preparation of fibrin hydrogels encapsulated with chondrocytes

For the preparation of fibrin-based hydrogels, human fibrinogen (Millipore) was dissolved in 10 mM HEPES (Sigma) at pH 7.4 to obtain a 50 mg/mL human fibrinogen solution, and human thrombin (Millipore) was dissolved in 10 mM HEPES at pH 6.5 supplemented with 0.1% BSA to obtain a 2 U/mL thrombin solution. A sodium-calcium chloride solution was also prepared with 3 M NaCl and 0.4 M CaCl_2_ in 10 mM HEPES at pH 7.4. A cell-fibrin suspension was prepared by mixing HNCs (resuspended in DMEM/F-12) with the fibrinogen solution, followed by the addition of thrombin and NaCl/CaCl_2_ to reach final concentrations of 10 mg/mL fibrinogen, 0.4 U/mL thrombin, 150 mM NaCl, 20 mM CaCl_2_ and 2 × 10^6^ HNCs. Solution volumes were adapted to the size of the scaffolds.

### Assembly of silicone scaffold-fibrin hydrogel constructs and culture in chondrogenic medium

To encapsulate a 10 mm^2^ printed scaffold, 500 µL of the chondrocyte-fibrin suspension was first dispensed into a stainless steel mold. The scaffold was then placed into the mold and bathed in the suspension, followed by the addition of another 500 μL to ensure complete coverage. The constructs were allowed to polymerize at 37°C for 1 h. After gelation, the molds were carefully removed, and the embedded constructs were transferred to a 12-well culture plate. Each well was filled with 1 mL of chondrogenic culture medium consisting of 1 × ITS (insulin, transferrin and selenium; Gibco), 50 µg/mL 2-phospho-L-ascorbic acid (trisodium salt; Fluka), 200 ng/mL recombinant human bone morphogenic protein (BMP-2) (Dibotermine-α, from the InductOs kit, Wyeth), 5 μg/mL insulin and 100 nM triiodothyronine (T3; Sigma). This combination of BMP-2, insulin and T3 is referred to as the BIT cocktail [11]. Culture medium was replaced three times per week for a total of 21 days.

For anatomically-sized nasal septum implants, 2 mL of chondrocyte-fibrin gel was injected into each injection port of the mold containing the full-size scaffold, for a total of 4 mL. After 1 h of polymerization at 37°C, the constructs were carefully demolded and cultured in the chondrogenic BIT medium for 21 days under gentle orbital shaking, with medium changes three times per week.

The morphology and macroscopic appearance of the printed scaffolds and full-scale nasal scaffolds were observed before and after the addition of the cellularized gel using a Leica M80 binocular loupe, with LAS software in brightfield mode and an IC80HD camera.

### Gene expression analysis

Following *in vitro* culture, segments of tissue-engineered constructs were recovered and the silicone fragments were discarded. The remaining hydrogel was cut using a scalpel, then frozen in liquid nitrogen and ground using a mortar and pestle. Total RNA was isolated using the Nucleospin RNA II kit (Macherey-Nagel), according to the manufacturer’s instructions. Reverse transcription was performed as previously described [14]. Real-time polymerase chain reaction amplification was carried out on a Rotor-Gene Q cycler (Qiagen) using the FastStart Universal SYBR Green Master (Roche), as previously described [17]. Primer sequences, along with accession numbers or corresponding references [14, 19, 20], are listed in Table 2. Results are expressed as relative expression values normalized to the ribosomal protein L13a gene (*RPL13a*) and quantified using the comparative Ct method. Each measure was performed in duplicate.

**Table 2:** Oligonucleotide primers used for the real-time polymerase chain reaction analyses.^a^

Gene	Sequence (5′→3′)	Reference
*RPL13A*	Forward: AAGGCATCAACATTTCTGGCAA Reverse: GGGTTGGTGTTCATCCGCTT	*NM_012423.4*
*COL9A1*	Forward: ACGGTTTGCCTGGAGCTAT Reverse: ACCGTCTCGGCCATTTCT	[13]
*COL2A1*	Forward: TCCATGTTGCAGAAAACCTTCA Reverse: GGAAGAGTGGAGACTACTGGATTGAC	[18]
*ACAN*	Forward: TCGAGGACAGCGAGGCC Reverse: TCGAGGGTGTAGCGTGTAGAGA	[19]

a Primers are presented in a 5′–3′ orientation. The source of the databank used for designing the primers is presented as an accession number. When primers have been used in other studies, the references are indicated.

### Western blot analysis

Following *in vitro* culture or explantation from mice, segments of tissue-engineered constructs were cut using a scalpel, frozen in liquid nitrogen and ground with a mortar and pestle. Silicone fragments were discarded and 100 µL of the fibrin-based gels were resuspended and boiled in 300 µL of 2× Laemmli buffer containing 3% β-mercaptoethanol. Twenty microliters (20 µL) of each sample were separated by sodium dodecyl sulfate-polyacrylamide gel electrophoresis (SDS-PAGE) on 4–12% gradient gels and transferred to polyvinylidene difluoride (PVDF) membranes (Millipore) using CAPS buffer (N-cyclohexyl-3-aminopropanesulfonic acid, Sigma) supplemented with 7.5% methanol. After transfer, membranes were probed with primary antibodies, washed and then incubated with secondary antibodies (Table 3). After several washes, bound antibodies were detected on X-ray films using Immun-Star alkaline phosphatase or horseradish peroxidase (HRP) chemiluminescent substrate (Bio-Rad). When re-probing was required, membranes were stripped using ReBlot Plus Strong solution (Millipore).

**Table 3:** List of primary and secondary antibodies used for Western blotting and immunofluorescence analyses.^a^

	Antibodies	Dilution and application	Reference and supplier
Primary antibody	Anti-type II collagen Rabbit polyclonal	1:2500 (WB)	Novotec (Ref 20211)
	Anti-type II collagen Mouse monoclonal (clone 6B3)	1:100 (IF)	Millipore (Ref MAB8887)
	Anti-type IX collagen Mouse monoclonal (clone 23-5D1)	1:3000 (WB)	Millipore (Ref MAB3304)
	Anti-Sox9 Rabbit polyclonal	1:3000 (WB) 1:100 (IF)	Millipore (Ref AB5535)
	Anti-Actin Rabbit polyclonal	1:3000 (WB)	Sigma (Ref A2066)
Secondary antibody	AP-conjugated anti-rabbit	1:3000 (WB)	Cell Signaling Technology (Ref 05/2016)
	HRP-conjugated anti-mouse	1:3000 (WB)	Cell Signaling Technology (Ref 11/2010)
	Alexa Fluor 546-conjugated anti-mouse	1:500 (IF)	Invitrogen (Ref A11030)
	Alexa Fluor 488-conjugated anti-mouse	1:500 (IF)	Invitrogen (Ref A11034)

a AP = alkaline phosphatase; IF = immunofluorescence; WB = Western blotting.

### Immunofluorescence analysis

Immunofluorescence analysis was performed on entire constructs or on construct segments approximately 0.2 cm in cross-section and 1 cm in length, cut using a scalpel and placed into microscope plate wells (µ-Plate 24 Well Black, ID 14 mm, Ibidi). Samples (whole constructs or segments) were fixed for 24 h in formol-acetic alcohol (AFA, Microtech), then permeabilized with 0.1% Triton (Triton X-100, Sigma) for 20 min at RT. After washing in PBS, the samples were incubated for 45 min at RT in 1% bovine serum albumin (BSA, Sigma) in PBS (BSA/PBS), followed by overnight incubation at 4°C with primary antibodies (Table 3) diluted in BSA/PBS. After several PBS washes, samples were incubated for 1 h at RT with secondary antibodies (Table 3) diluted in BSA/PBS. Nuclear staining was performed using 1X Hoechst solution (Fluka) in PBS for 5 min at RT. For viability analysis, simultaneous fluorescence staining of live and dead cells was performed using the Cellstain Double Staining Kit (Sigma-Aldrich), according to the manufacturer’s instructions. All fluorescence images were acquired using an Eclipse Ti-E microscope (Nikon) equipped with either a color (DS-Fi2) or a monochrome (DS-Qi2-ND) camera and the NIS-Elements imaging software.

### Safranin-O and Alcian blue staining of chondrocyte-fibrin hydrogels

Following fixation, fibrin-silicone constructs were dehydrated in a series of graded ethanol baths (30% ethanol for 10 min, 50% ethanol for 30 min, 70% ethanol for 30 min, 95% ethanol for 2 h and 100% ethanol for 2 h). Samples were embedded in paraffin using a Histo 5 automated tissue processor (Milestone Medical) and sectioned with a 3 µm thickness using a microtome. Paraffin was removed by immersion in a 3:1 (v/v) butanol/methylcyclohexane mixture for 2 h, then a 1:3 (v/v) butanol/methylcyclohexane mixture for 2 h and finally pure methylcyclohexane for 4 h. Sections were collected, mounted on glass slides and hydrated with distilled water before staining. For hematoxylin staining, sections were dipped in a solution of Hematoxylin QS Solution (Vector Laboratories, Inc) for 5 min, washed with water for 5 min, dipped three times in a 2:5 mixture of 36% HCl:70% EtOH and washed with water twice for 1 min. To detect glycosaminoglycans (GAGs), sections were stained with either 0.1% Safranin-O in distilled water (pH 7.4) for 5 min, or with 0.1% Alcian blue in 3% acetic acid (pH 2.6) for 20 min. Sections were then washed with water, 96% ethanol twice and xylene twice for 2 min. Image acquisition were performed on an Eclipse Ti-E microscope (Nikon).

### In vivo experimentation

*In vivo* experiments were performed in strict accordance with the official regulation on animal experimentation (Directive 2010/63/EU and its national transposition) and ethical guidelines for care and use of mice of the Plateau de Biologie Expérimentale de la Souris (Mouse Experimental Biology Platform, UMS 3444) at Ecole Normale Supérieure (ENS, Lyon). The *in vivo* study was approved by the Committee on the Ethics of Animal Experiments of ENS de Lyon (approval number: ENS_2019_005). Mice were maintained in the Animal Care Facilities of PBES under pathogen-free conditions with food and water *ad libitum*. The animals were regularly monitored by staff responsible for general animal health and welfare supervision. All surgeries were performed under general anesthesia and all efforts were made to minimize suffering. Female nude mice (6 weeks old) were obtained from Charles River Laboratories. Animals were anesthetized with isoflurane gas, and surgeries were performed under a laminar flow hood in sterile conditions. Two subcutaneous pockets were created on the back of each mouse by blunt dissection. Centimetric silicone models coated with fibrin gel containing chondrocytes were inserted into the pockets and the skin was closed with surgical staples (Harvard Apparatus, ref 52–3746). After 6 weeks, the mice were euthanized by cervical dislocation and the silicone-fibrin constructs were explanted.

### Dynamic mechanical analysis

The viscoelastic behavior of human nasal septum, silicone scaffold and engineered nasal septum (made from HNCs encapsulated in fibrin cast around a silicone scaffold) was characterized by frequency sweep experiments ranging from 0.1 to 10 Hz, conducted in flexural mode using dynamic mechanical analysis (DMA) equipped with a 10 mm frame. For fibrin alone, experiments in flexural mode could not be carried out due to the softness of the hydrogel, and a shear test with a 2.5% strain at a frequency of 1 Hz on an 8 mm-wide geometry was done instead. Measurements were performed on a stress-controlled rheometer (Discovery Hybrid Rheometer 2, TA Instruments, USA). Prior to measurements, the linear viscoelastic region was determined via an amplitude sweep at 1 Hz. The experimental data were fitted using a generalized Maxwell model with three elements, as previously described [21].

### Surgical testing

A surgical test was carried out on the cadaver of a 77-year-old man in the anatomy laboratory of the Lyon Est medical school (Rockefeller campus), in accordance with the French bioethics law of 2 August 2021, which governs body donation, and the laboratory’s ethical charter on respect for the cadaver. An open surgical approach to the nasal septum in cadavers was performed using standard rhinoseptoplasty instruments. Subperichondrial dissection of the cartilaginous septum was performed, preserving the nasal mucosa on both sides. The entire septal cartilage was removed, while the lower lateral and upper lateral cartilages were preserved. An osteotomy of the nasal dorsum was performed to create an open-top bony notch at the level of the nasal bones. After removal of the native septum, anatomically-sized implants made from chondrocyte-loaded fibrin gels cast around full-sized 3D-printed scaffolds and cultured for 3 weeks as described above were implanted under surgical conditions. The implant was inserted while preserving the anatomical relationships between the septal cartilage, maxilla and nasal bones. The skin, as well as the alar and triangular cartilages, was repositioned over the septum prosthesis to allow postoperative analysis.

### Statistical analysis

Reported values are mean ± standard deviation, obtained from 3 to 5 independent biological repeats (*N* = 3, 4 or 5). A different donor was used for each biological repeat. Statistical analysis was carried out using Prism software (GraphPad, San Diego). Legend on figures **** denotes *P* < 0.0001, *** denotes *P* < 0.001 ** denotes *P* < 0.01, * denotes *P* < 0.05 and ns denotes non-significant.

## Results

### Experimental design

**Figure 1:**
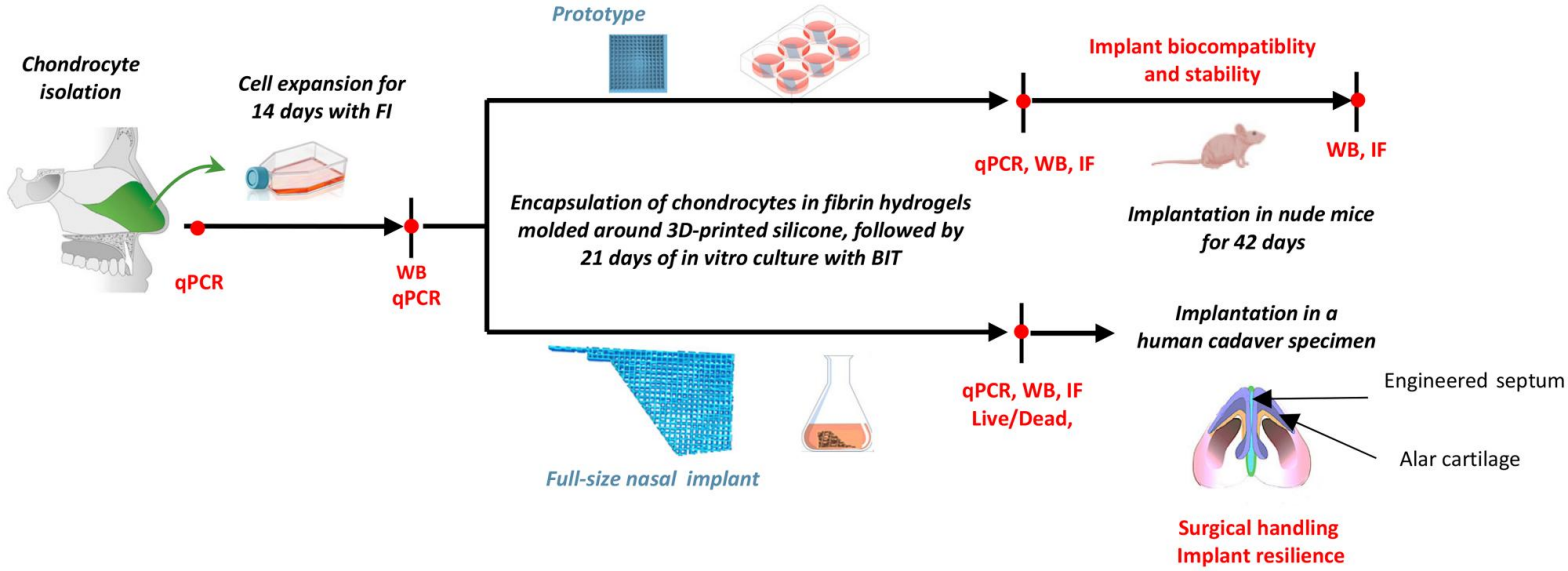
Experimental design schematic. Each dot shows the time points of analyses.

About 2 × 10^5^ to 1 × 10^6^ viable HNCs were routinely extracted from sections of the nasal cartilage weighing as little as 0.2 g. HCNs were expanded in 2D tissue culture plates in the presence of FGF-2 and insulin to stimulate proliferation, up to sub-confluency to obtain enough cells (8 × 10^6^ cells) to generate full-sized nasal implants. During this expansion step, HNC de-differentiate and subsequently require to be re-differentiated into mature chondrocytes [12, 13, 22] in a 3D environment and in the presence of growth factors that include BMP-2 and T3. Here, expanded HNCs were encapsulated in fibrin hydrogels and treated with the BIT cocktail. HNC-loaded fibrin gels were cast around 3D-printed silicone porous scaffolds, either of a prototype shape (10 mm × 10 mm × 2 mm) designed for *in vivo* experimentation in mice (donors 1–10), or in the shape of a full-sized nasal septum implant to assess its applicability in surgery (donors 11–18). A schematic of the experimental setup is shown in Figure 1.

### HNCs show efficient re-differentiation when embedded in fibrin hydrogels cultured within a silicone-based prototype model

**Figure 2:**
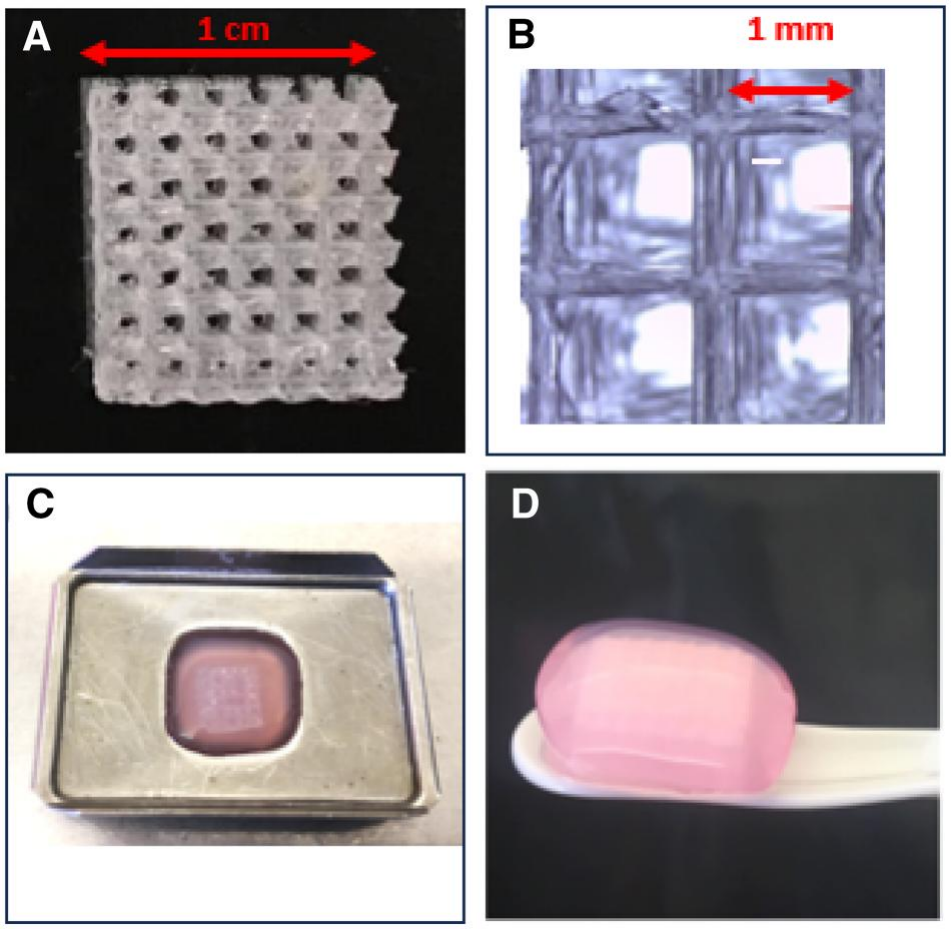
Macroscopic images of the 3D-printed silicone scaffold prototype. (**A**) The silicone prototype was printed in a square shape, with external dimensions of 10 mm per side and a thickness of 2 mm. (**B**) The scaffold features square pores measuring 1 mm across. (**C**) Top view of a prototype placed in a stainless steel mold for paraffin embedding, covered with a fibrin gel containing HNCs. (**D**) After polymerization of the gel, the construct was carefully removed from the mold and handled with a spatula.

In a preliminary set of experiments, 3D-printed silicone scaffold prototypes (10 mm × 10 mm × 2 mm) were 3D-printed with pores within its structure (Supporting Information 1). A porosity of 60% and a pore size of 1 mm enabled the fabrication of a 3 mm-thick scaffold without collapsing of the pores during the 3D-printing process. This led to pores remaining open on both side of the scaffold, which is a prerequisite for fibrin gel to fill in the pores upon injection of the fibrinogen/thrombin mixture. With this geometry, chondrocyte-laden fibrin was cast around and within the 3D-printed porous scaffolds, allowing for anchoring of the gels to the silicone frame (Figure 2A–D).

**Figure 3:**
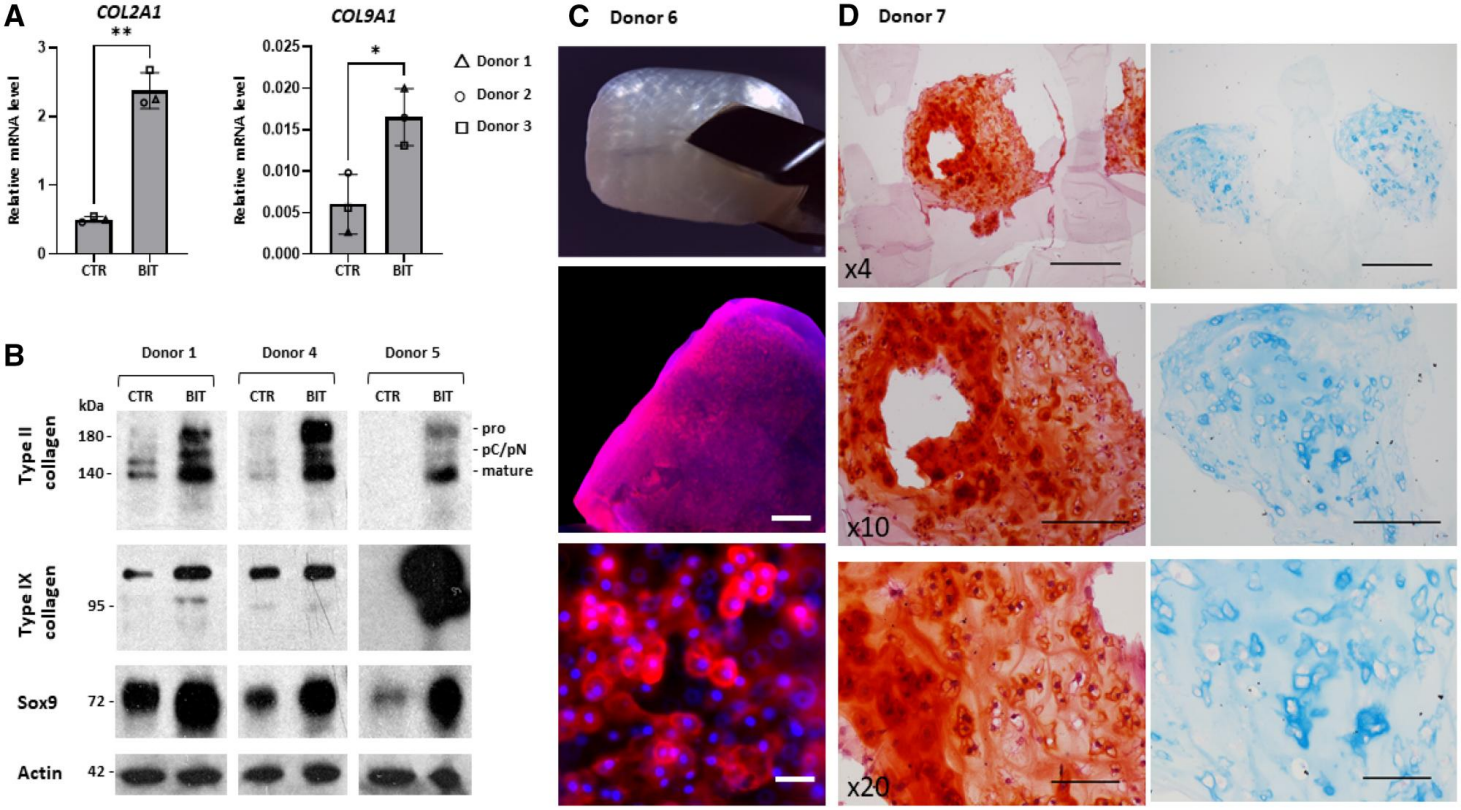
Treatment with BIT promotes cartilage matrix reconstruction in fibrin hydrogel. HNCs were expanded for 2 weeks on plastic in the presence of 10% FBS supplemented with FI, encapsulated in fibrin cast around silicone scaffolds and cultured for 21 days *in vitro*, either in control medium with no growth factors (CTR) or in chondrogenic medium containing BIT. (**A**) Gene expression analysis of *COL2A1* and *COL9A1*. Values are presented as mean ± SD. Statistical analysis was performed using paired *t*-tests (*N* = 3). ** denotes *P* < 0.01; * denotes *P* < 0.05. (**B**) Western blot analysis of type II and type IX collagens, and Sox9 (*N* = 3). The positions of mature collagen chains (mature), unprocessed precursors (pro) and processing intermediates containing the carboxypropetide (pC) or aminopropeptide (pN) are indicated. (**C**) Type II collagen immunofluorescence staining of a prototype scaffold after 21 days of *in vitro* culture in the presence of BIT. Top: whole construct before staining. Middle: immunostaining on the construct showing strong type II collagen labeling (red) in fibrin around the scaffold (scale bar = 200 µm). Bottom: high-magnification view showing abundant type II collagen synthesis by the chondrocytes. Nuclei were stained with Hoechst (blue) (scale bar = 20 µm). (**D**) Safranin-O/hematoxylin (left) and Alcian blue (right) staining of paraffin sections of fibrin/silicone scaffold constructs cultured with chondrocytes for 21 days *in vitro* in the presence of BIT, showing abundant GAG and proteoglycan deposition (scale bar: ×4 = 500 µm, ×10 = 250 µm, ×20 = 100 µm).

Following expansion on plastic, the ability of HNCs from donors 1 to 10, embedded in fibrin and cast around and within the silicone scaffold prototype, to respond to the BIT cocktail was assessed after a 21-day culture period. Gene expression analysis revealed that BIT-treated cells exhibited significantly higher levels of mRNA transcripts for *COL2A1* and *COL9A1*, two genes encoding key cartilage matrix collagens, compared to the control conditions with no growth factor, for all three tested donors (Figure 3A). Western blot analysis in three independent donors confirmed the enhanced synthesis of type II and type IX collagens upon BIT treatment, compared to untreated expanded HNCs (especially for donors 1 and 5 for type IX collagen). Notably, type II collagen was detected in its unprocessed, intermediate and mature forms, which indicates active matrix production and tissue maturation. Additionally, BIT strongly upregulated Sox9, a master transcription factor of chondrogenesis, in all donors (Figure 3B). Immunofluorescence analysis supported these findings by showing intense type II collagen staining within the gel, particularly around the chondrocytes (Figure 3C), confirming the cartilage ECM production activity of HNCs. This trend was further confirmed by Safranin-O and Alcian blue staining, demonstrating GAG and proteoglycan deposition across the hydrogel (Figure 3D). Immunofluorescence also showed a homogeneous distribution of HNCs within fibrin hydrogels, and a qualitatively similar type II deposition on the edges of the scaffold and within pores. Overall, these results indicate that HNCs efficiently re-differentiated into ECM-producing chondrocytes upon BIT treatment, following encapsulation in fibrin anchored to silicone-based scaffolds.

### Stability of the neo-synthesized matrix following subcutaneous implantation

**Figure 4:**
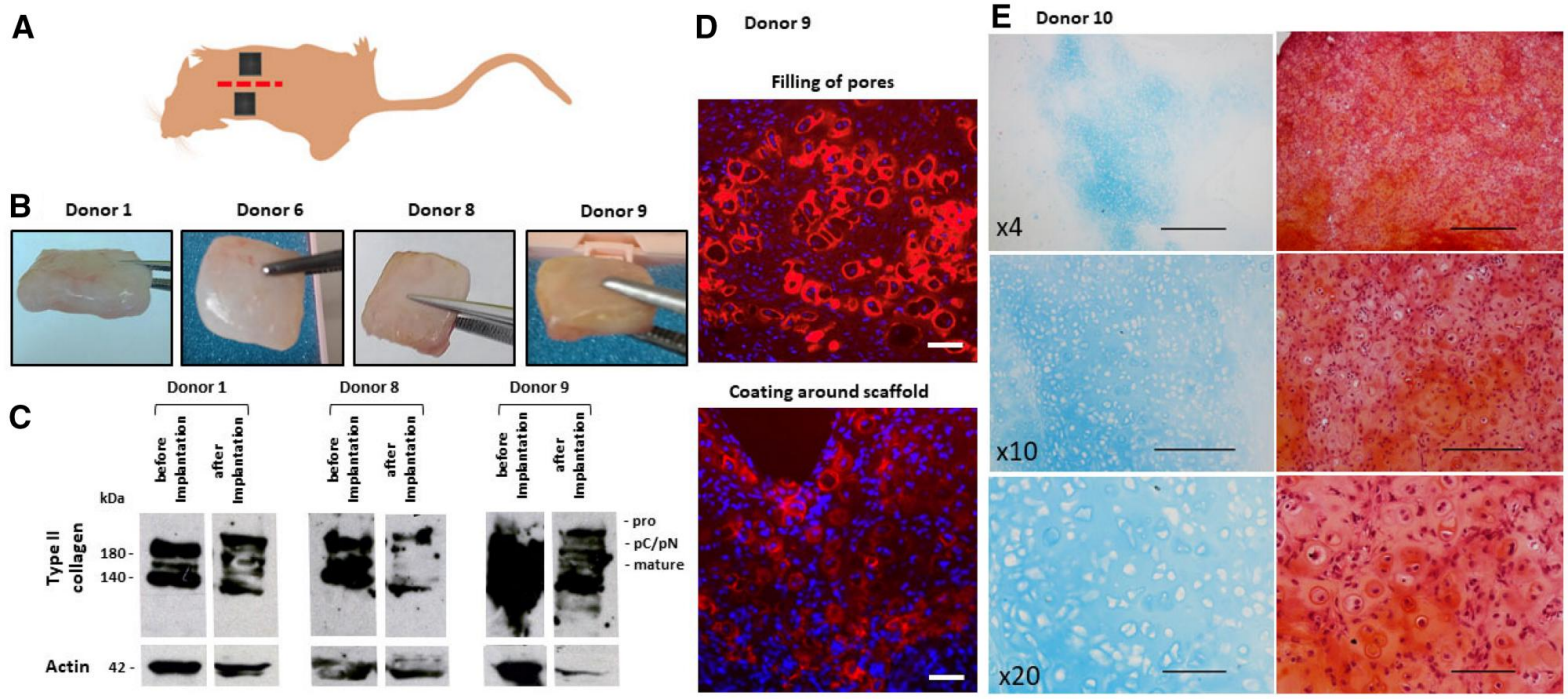
Stability of the engineered constructs after subcutaneous implantation in nude mice. (**A**) Schematic representation of the implantation sites per animal (black squares). Two prototype constructs were implanted subcutaneously in the back of each mouse. (**B**) Macroscopic appearance of constructs harvested 42 days post-implantation, showing preservation of their structural integrity. (**C**) Western blot analysis of type II collagen in segments of the same constructs analysed before implantation (after 21 days of *in vitro* culture with BIT) and 42 days post-implantation (*N* = 3). The presence of unprocessed, intermediate and mature collagen forms is maintained. (**D**) Immunofluorescence staining for type II collagen (red) performed on a construct harvested 42 days post-implantation, showing a collagen-rich matrix surrounding the chondrocytes. Nuclei were stained with Hoechst (blue) (scale bar = 20 µm). (**E**) Alcian blue (left) and Safranin-O/hematoxylin (right) staining of sections of a silicone-fibrin construct harvested 42 days post-implantation, showing GAG and proteoglycan deposition across the fibrin hydrogel (scale bar: ×4 = 500 µm, ×10 = 250 µm, ×20 = 100 µm).

To assess the *in vivo* behavior of re-differentiated HNCs and the stability of the neo-synthesized matrix within silicone scaffolds, constructs with HNCs from four donors were cultured for 21 days with BIT and were implanted subcutaneously in the backs of nude mice, exposing them to skin tension stress (Figure 4A). After 42 days, the mice were euthanized and gross examination of the explanted constructs revealed that they retained their structural integrity (Figure 4B).

Western blot analysis of the gel confirmed that the presence of type II collagen was maintained for all three donors, including unprocessed, intermediate and mature forms, similar to those observed prior to implantation (Figure 4C). This suggests that the neo-formed cartilage gel remained stable and that transplanted chondrocytes continued to contribute metabolically to matrix maintenance. Immunofluorescence analysis validated the presence of a type II collagen-rich matrix around the chondrocytes (Figure 4D), both located inside the pores of the silicone scaffold, and on the edges of the construct. Histological staining showing GAG and proteoglycan deposition, assessed by Safranin-O and Alcian blue staining (Figure 4E), further supported the presence of a sustained cartilage ECM in the construct 42 days after implantation in mice.

### Manufacture of the nasal septum implant

**Figure 5:**
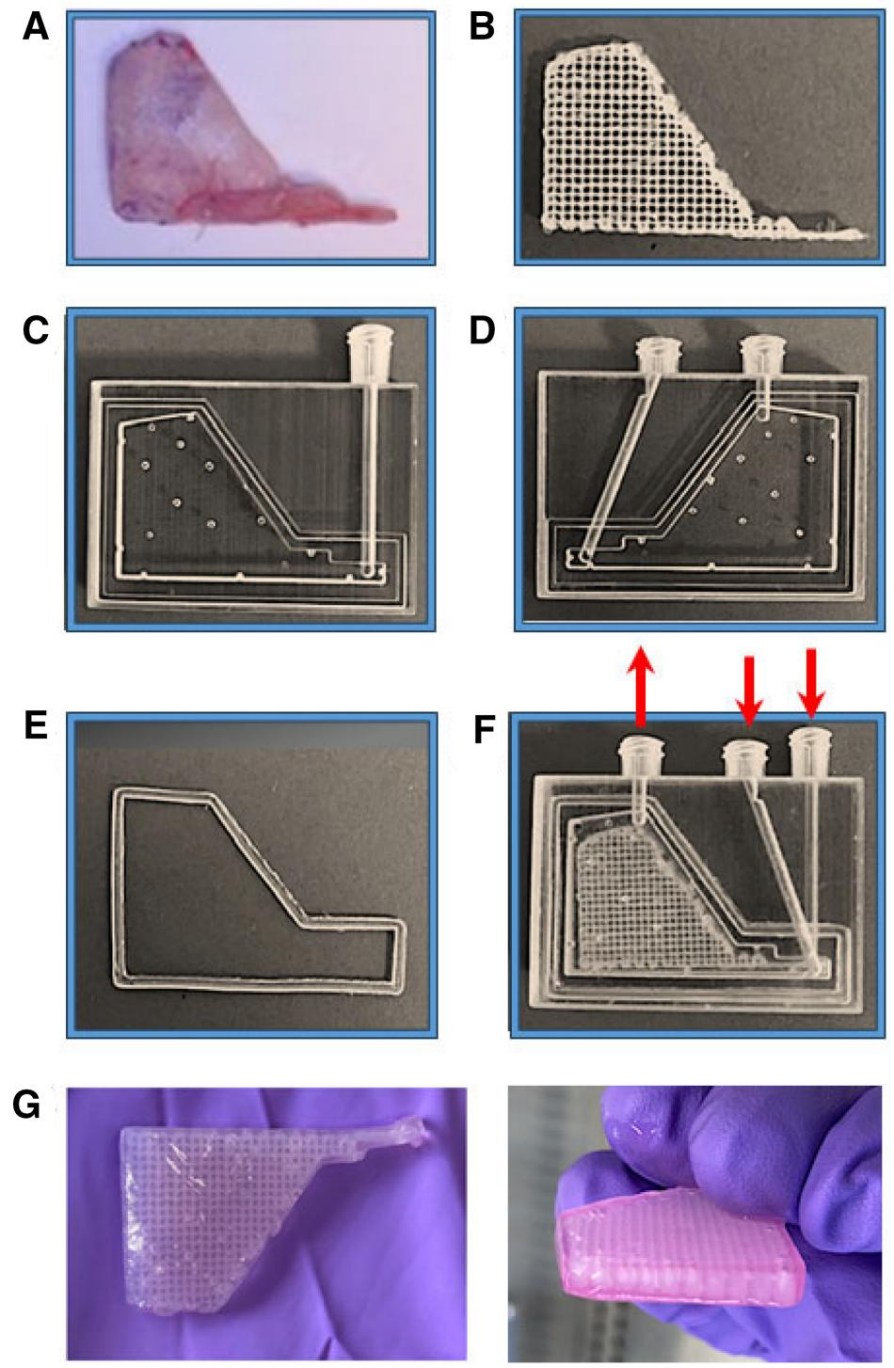
Photographs of the 3D-printed nasal scaffold and the cover mold. (**A**) L-shaped nasal cartilage structure, called L-strut, assembled from fragments of human cartilage. L-struts are surgically implanted instead of the nasal septum to restore the native shape and respiratory function of the nose. (**B**) 1:1 scale porous silicone framework based on an L-strut. (**C**) Half of the mold with an injection tunnel. (**D**) Other half of the mold with both injection and ejection tunnels. The scaffold is held in the center of the mold by a system of very fine pins that elevate it on both sides, creating a 1 mm gap. (**E**) Silicone gasket aligning and sealing the two mold halves. (**F**) Scaffold positioned inside the closed mold. Arrows indicate the directions of injection and ejection flows. (**G**) Full-size septal implant after removal from the mold, covered with HNC-fibrin gel.

Next, we explored the possibility of scaling-up our methodology to a full-size nasal septum implant. The dimensions of the nasal implant were based on the L-shaped cartilage structure (known as the L-strut, Figure 5A) that is usually reconstructed from fragments of rib or ear cartilage by surgeons, when the septal cartilage was severely deformed or destroyed [6]. A porous scaffold model measuring 40 mm × 20 mm in a L-shape was fabricated from silicone by 3D printing (Figure 5B). To homogeneously cast fibrin gels around such scaffolds, cover molds consisting of two parts were designed, one containing an injection port and the other containing both an injection port and an outlet port (Figure 5C and D). The mold cavity featured a 1 mm offset volume that extended over each side of the implant model. The scaffold was placed in one part of the cover mold and the other part was pressed into place, with a gasket surrounding the scaffold (Figure 5E and F). No leakage was observed when the fibrin gel loaded with HNCs was injected simultaneously using two syringes, each containing 2 mL of the gel mixture. The nasal implants were successfully demolded and macroscopic observation showed a scaffold adequately covered by the fibrin-HNCs mixture (Figure 5G).

### Preparation of engineered nasal cartilage in vitro

**Figure 6:**
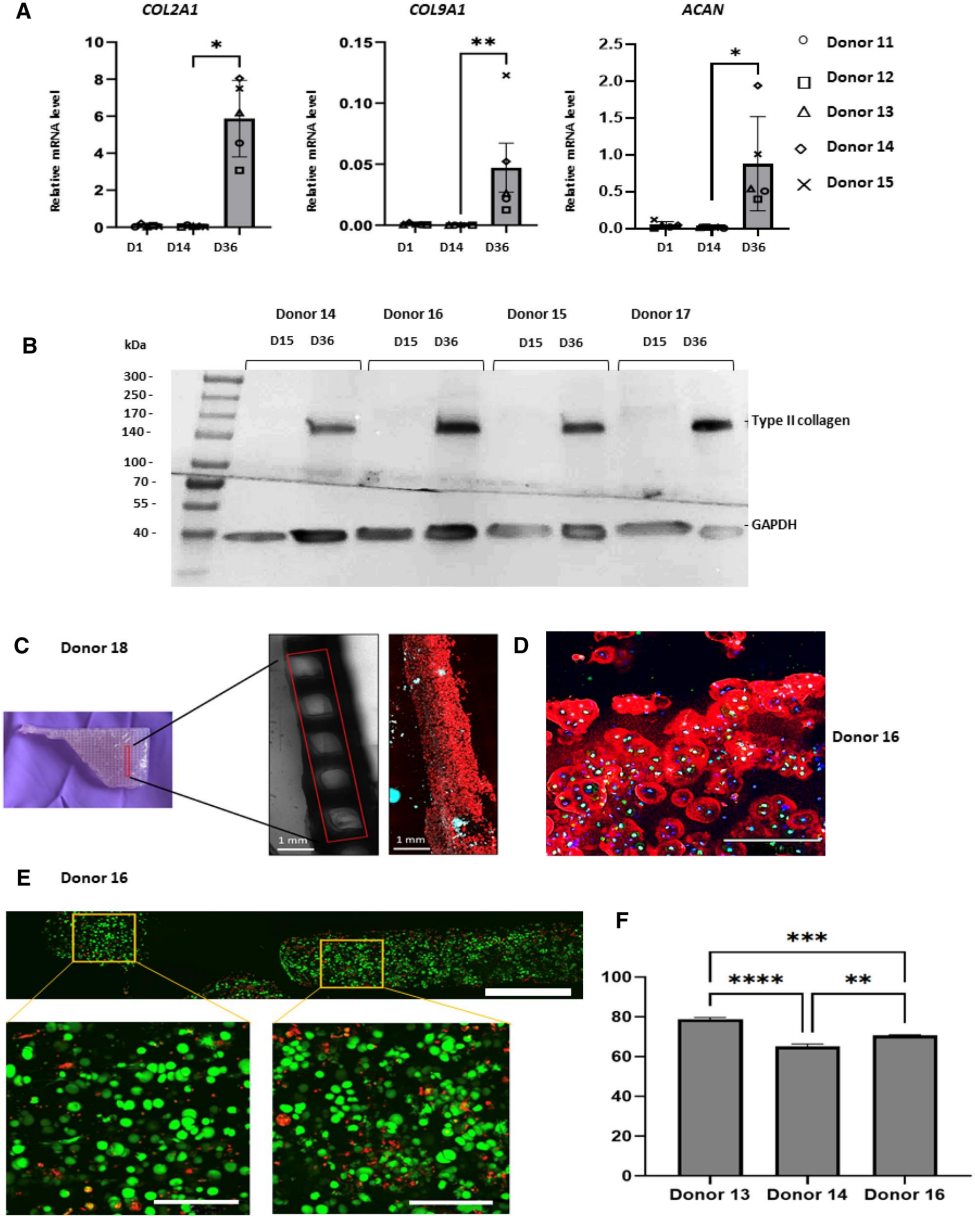
*In vitro* chondrogenic differentiation of full-size nasal cartilage constructs. HNCs were isolated and expanded on plastic for 2 weeks, then embedded in a chondrocyte-fibrin suspension and injected into a mold containing a full-scale L-shaped scaffold. After removal from the mold, the constructs were cultured *in vitro* for 21 days (3 weeks) in the presence of the BIT cocktail. (**A**) Relative mRNA expression was analysed at day 1 and day 14 of the expansion phase, and at day 36 following *in vitro* maturation. Statistical analysis was performed using Friedman tests followed by Dunn’s multiple comparisons *post hoc* test (*N* = 5). *P < 0.05; **P < 0.01. (**B**) Western blot analysis of type II collagen production in construct segments collected after 1 day (day 15) and at the end (day 36) of the *in vitro* maturation phase (*N* = 4). (**C**) Top view of a construct segment at day 36. Left: brightfield image. Right: Immunofluorescence staining for type II collagen in the same region. Image is a mosaic reconstructed from 10 confocal fields (scale bars = 1000 µm). (**D**) High-magnification view of type II collagen staining at the surface of a construct segment (day 36). Nuclei stained with Hoechst (blue); most nuclei appear indigo due to co-staining with Sox9 (scale bar = 40 µm). (**E**) Top view of live/dead staining of a construct segment harvested at day 36. Live cells are in green; dead cells are in red. Top: black zone indicates silicone scaffold (scale bar = 1500 µm). Bottom: high magnification of two selected areas (scale bars = 200 µm). (**F**) Quantification of cell viability in constructs from three independent donors. Statistical analysis was performed using one-way ANOVA followed by Tukey’s multiple comparisons *post hoc* test (*N* = 4). **P < 0.01. ***P < 0.001. ****P < 0.0001.

Having established the hydrogel injection technique, we evaluated its potential to promote chondrogenesis in a full-size nasal implant. First, HNCs from donors 11 to 18 were extracted (at D1) and expanded for 2 weeks (up to D14). This was accompanied by a slight, but not significant, decrease in *Col2A1*, *Col9A1* and *ACAN* (encoding for aggrecan, a cartilage-specific proteoglycan) expression, assessed by PCR in five donors, confirming that HNCs de-differentiate during the expansion step [12, 13, 22]. After HNC encapsulation in fibrin cast around the silicone scaffold and demolding, the nasal constructs were cultured for 21 days in the presence of BIT (up to Day 36). Gene expression analysis for constructs from five donors confirmed chondrocyte re-differentiation in the engineered nasal cartilage matured *in vitro*, as evidenced by the upregulation of *COL2A1*, *COL9A1* and *ACAN* expression (Figure 6A). Of note, levels of mRNA transcripts substantially varied between repeats, indicating variability between donors. Nevertheless, the expression of these chondrogenic markers were consistently higher after 3 weeks of culture in fibrin gels (Day 36) than for both freshly extracted (Day 1) and expanded (Day 14) HNCs. This highlights the efficiency of the BIT treatment regardless of the patient’s background. Western blot analysis confirmed that construct maturation was associated with robust synthesis of type II collagen in the four tested donors (Figure 6B). Our findings were further supported by immunofluorescence staining, which revealed the abundance of type II collagen in the constructs (Figure 6C). High-magnification imaging showed both extracellular and intracellular staining, indicating type II collagen deposition in the ECM and ongoing metabolic activity. This was corroborated by nuclear Sox9 immunostaining (Figure 6D). Consistent with this metabolic activity, a live/dead fluorescent assay revealed a donor-dependent cell viability ranging from 65% to 79%, measured across constructs from three donors after 3 weeks of culture (Figure 6E and F). Live and dead cells were homogeneously distributed throughout the fibrin hydrogels, with no apparent differences between the edges and the center of the gel (additional large fields of view of live/dead assays performed on engineered constructs are available in Supporting Information 2). In addition, no increase in cell death between 7 and 21 days of culture was observed, indicating that fibrin gels were compatible with HNC culture over 3 weeks (Supporting Information 2).

### Mechanical properties of in vitro engineered nasal cartilage

**Figure 7:**
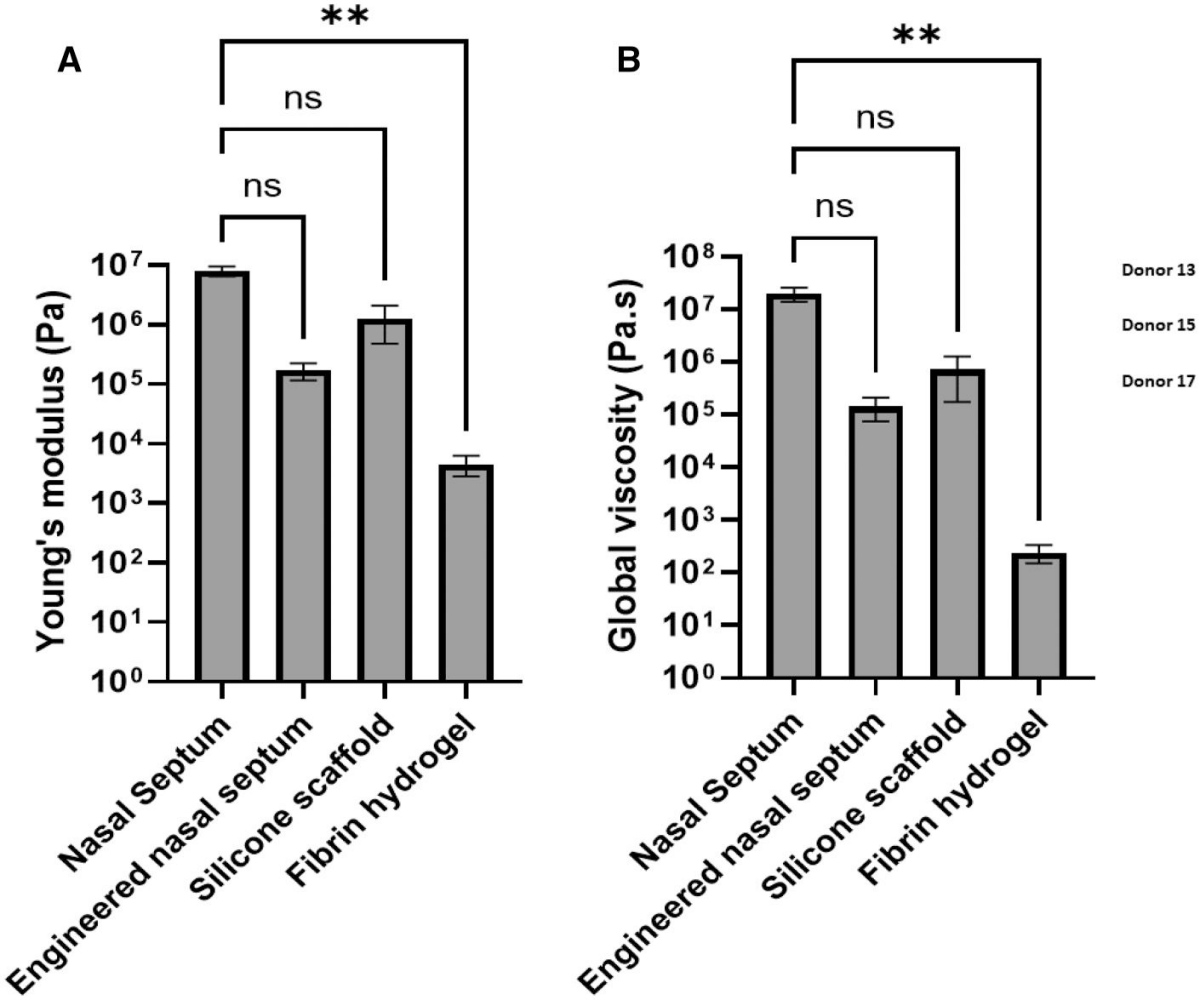
Comparison of the viscoelastic properties of fibrin hydrogels, human nasal septum, engineered nasal constructs and uncoated silicone scaffolds. Three independent samples were analysed per group. Human nasal septa were obtained from donors distinct from those whose cells were used to fabricate the engineered constructs. (**A**) Young’s modulus, representing the elastic stiffness of the material (*N* = 3). (**B**) Global viscosity, reflecting time-dependent viscoelastic behavior (*N* = 3). Statistical analysis was performed using Kruskal–Wallis tests followed by Dunn’s multiple comparisons *post hoc* test. **P < 0.01.

Dynamic mechanical analyses were performed to compare the viscoelastic properties of four groups of samples: human nasal septa, fibrin hydrogels, full-scale uncoated silicone scaffolds and full-scale engineered nasal constructs. Samples were characterized by both their elastic (Young’s modulus) and viscous (global viscosity) components. Native septal cartilage exhibited superior Young’s modulus and global viscosity, presumably due to its highly organized ECM, enriched with collagen fibers and proteoglycans, which confer both energy dissipation capacity and mechanical resistance. The uncoated silicone scaffolds exhibited a roughly 1-log reduction in both Young’s modulus and global viscosity compared to the native human nasal septum, while the engineered nasal constructs showed a 2-log reduction (Figure 7). However, this decrease was not statistically significant. By contrast, fibrin hydrogels had a significantly lower Young’s modulus and global viscosity than that of native nasal septum (with a 3-log and 5-log decrease, respectively).

### Surgical test

The nasal implant was evaluated for its manipulability and mechanical resistance during handling by a surgeon specializing in rhinoseptoplasty. Using standard surgical instruments, the surgeon ensured that no damage occurred to the coated cell-laden fibrin gel (Supporting Information 3A–D). The implant’s dimensions were validated in three directions to confirm its ability to restore nasal length and projection while maintaining patency. This was assessed through palpation, clinical examination and photographic analysis (Supporting Information 3E and F). Furthermore, the implant’s mechanical strength was tested for its capacity to withstand skin tension without compromising the surrounding tissues. Palpation confirmed the implant’s robustness and its suitability for clinical application.

## Discussion

In this study, we explored the design, fabrication and implantation of a nasal implant constructed from medical-grade 3D-printed silicone and fibrin hydrogel loaded with HNCs. Its structural integration and implantation feasibility were investigated respectively through small-scale *in vivo* trials in nude mice and full-scale anatomical fitting in a human cadaver. While polycaprolactone is the most popular material for nasal cartilage engineering [23–33], we chose instead a 3D-printable silicone scaffold for its increased durability, flexibility and elasticity. Silicone was also selected for its excellent printability, allowing precise fabrication of custom implants. From a biomechanical perspective, fibrin hydrogels alone were dramatically softer than native human nasal septa, indicating that their use on their own is not suitable for nasal cartilage repair. While fibrin stiffness can be enhanced through crosslinking with genipin [34], a natural cyto-compatible crosslinking agent [35, 36], we chose instead to combine fibrin with a silicone scaffold to provide overall mechanical stiffness and structure. This enabled the final engineered constructs to display viscoelastic properties that were not statistically different from those of native human septal cartilage.

Although silicone is generally considered biocompatible, its implantation in the nasal cavity is associated with a risk of chronic inflammation, rejection and superinfection, particularly due to the high microbial load and unique anatomical features of the nasal environment [37–40]. Bacterial infections, especially, represent a clinical challenge in bone or cartilage engineering, where biomaterials are typically designed to withstand heavy mechanical loads, but are often biologically inert. This leads to poor integration within the native tissue, resulting in misdirected or insufficient immune responses, allowing bacteria to colonize the implant surface and evade clearance. To address these limitations, we focused on enhancing the implant’s biocompatibility by incorporating a bioactive fibrin hydrogel loaded with HNCs. This strategy aims to promote better host tissue integration and reduce adverse immune responses following implantation. Fibrin has been successfully used in nasal cartilage engineering, either combined with decellularized ECM [34], or polycaprolactone [25]. Fibrin is also compatible with the incorporation of bioactive compounds, such as antimicrobial peptides, which we have previously shown to possess a strong antibacterial activity in our laboratory [41, 42].

Our initial results showed that a porous silicone structure offered a supportive environment for fibrin gels hosting HNCs, with an average of 71% cell viability after three weeks of culture. For some donors, over 30% cell death after 3 weeks of culture may represent an issue for future clinical applications. The homogeneous spatial distribution of live and dead cells suggests that the observed cell death was not due to the limited diffusion of oxygen, nutrients or growth factors. Instead, because viability did not decrease over time, we hypothesized that excess cell death may have occurred during the initial encapsulation process, in particular due to the shear stress exerted on cells during the perfusion of fibrin around the silicone scaffolds. The steady cell viability over 3 weeks tends to indicate that fibrin hydrogels remain compatible with the long-term culture of nasal chondrocytes.

Our study fits within the context of autologous chondrocyte transplantation, a technique initially developed for the treatment of knee articular cartilage defects [43]. In situations where nasal cartilage is unavailable, such as after trauma, repeated surgery or oncological resection, alternative cell sources must be considered. These include cartilage harvested from other anatomical sites, such as auricular or rib cartilage, which have already been used clinically in nasal reconstruction [44–46]. Alternatively, stem cell-based strategies, using mesenchymal stem cells (from adipose tissue [26, 47, 48], Wharton’s jelly [49] or bone marrow [50–52]) or iPSC-derived chondroprogenitors, have led to promising outcomes, replicating the chondrogenic profile of nasal chondrocytes in bioengineered nasal constructs. However, their use remains experimental. In parallel, clinical trials involving nasal septum-derived chondrocytes for nasal reconstruction are still in the early phases, with initial outcomes indicating restored alar lobule [53]. Notably, nasal chondrocytes exhibit superior proliferative and chondrogenic capacity compared to articular chondrocytes [9], motivating their use in focal articular cartilage repair [54]. These biological advantages make them particularly well-suited for engineering high-quality nasal cartilage. Our expansion and re-differentiation protocol are optimized for small biopsies, which is advantageous in clinical settings where donor tissue is limited. From as little as 0.2 g of nasal cartilage, enough viable HNCs could be extracted and expanded to generate a full-sized nasal implant using our injection molding approach.

Fibrin hydrogel effectively supported HNC re-differentiation following BIT treatment *in vitro*, as demonstrated by the expression of *COL2A1* and *COL9A1* genes (for prototypes and full-size constructs), as well as *Sox9* (for full-size constructs). Importantly, despite the inherent high donor variability associated with cells extracted from human tissues, results were significant and the same trends were observed regardless of the patient’s sex and age, highlighting the robustness of our approach. The formation of a cartilage-like tissue was also confirmed by an increase in type II collagen expression at the protein level across all donors, as well as in GAG and proteoglycan deposition. This is consistent with our previous results on the ability of fibrin to support chondrogenic re-differentiation in the presence of the BIT cocktail [16, 17]. The neo-cartilaginous tissue formed from expanded HNCs over the 3D-printed scaffold offers the key advantages of an autologous nasal graft, including minimal immunogenicity and improved integration with surrounding tissues, such as the nasal mucosa. Similar strategies have been explored in cartilage engineering. For instance, the biofunctionalization of 3D-printed silicone implants with immunomodulatory hydrogel coatings (gelatin loaded with IL-10 and PGE_2_) has been shown to reduce inflammation and improve survival in tracheal defect models, by promoting macrophage polarization toward a pro-healing M2 phenotype [55]. While such strategies aim to improve early host compatibility through immunomodulation, they often lack the capacity to recreate a tissue-specific microenvironment required for long-term structural and functional integration.

In contrast, our approach not only supports initial tolerance, through the use of a biocompatible fibrin-based hydrogel, but also seeks to rebuild a cartilage-like niche using autologous nasal chondrocytes. This biomimetic environment is expected to promote chondrocyte survival and matrix deposition, thereby facilitating progressive and durable integration with native nasal structures. A recent clinical trial for nasal septal perforation supports this concept, demonstrating that engineered autologous nasal cartilage grafts, combined with biological coatings, promote not only implant stability but also mucosal regeneration and integration, without requiring additional suturing of the nasal mucosa [56].

The transition from bench to surgical testing was explored by scaling-up our system to fabricate full-sized, anatomically accurate nasal septum constructs. We designed a nasal implant model that replicates the structure of an L-strut, which are constructed from autologous cartilage in conventional surgical approaches for the repair of the nasal septum. To cast fibrin homogeneously around scaffolds with such a geometry, a specific mold with injection tunnels were designed. Injection molding allowed us to reproducibly fill and cover the entirety of the 3D-printed silicone framework with fibrin-HNC gel, which was then matured into an engineered cartilage-like tissue *in vitro*. In addition, to ensure optimal biocompatibility, high cell viability and efficient ECM formation are necessary during the maturation phase prior to implantation. After 21 days of *in vitro* culture, these conditions were successfully met in the nasal septum constructs, as evidenced by the expression of genes encoding for markers of the cartilage tissue and the production of type II collagen.

*In vivo* testing for the tissue engineering of nasal septum is constrained by the lack of animal models that accurately replicate the human nasal framework [57]. Biomaterials for nasal septum repair have been implanted *in vivo* orthotopically in rabbits [24, 25, 50, 58], minipigs [23] or rats [59], but these models lack the protrusion and the thin overlying skin of the human nose. We thus chose to explore the structural integrity of our constructs through two avenues: the subcutaneous implantation in mice of prototypes (with a reduced size compatible with implantation in the back of the mouse), and the cadaveric testing of a full-scale nasal septum implant.

*In vivo* experiments in nude mice revealed that the bioengineered implants, when implanted subcutaneously, maintained their shape and integrity for 42 days. A homogeneous cartilaginous ECM (rich in type II collagen, proteoglycans and GAGs) was detected in the explanted structures, despite continuous skin pressure. This stability indicates that the implants can withstand mechanical forces and degradation over time, which is critical for their use in repair and nasal reconstruction procedures. This suggests that the 3D-printed silicone scaffold played a key role in ensuring *in vivo* mechanical stability. Crucially, no vascularization of the constructs was observed, contrary to previous studies combining fibrin hydrogels, polycaprolactone (which is non-resorbable similarly to our silicone scaffolds) and rabbit articular chondrocytes [25]. The presence of blood vessels in engineered cartilaginous tissue may indicate unwanted de-differentiation of chondrocytes, the formation of fibrocartilage or excessive inflammation. Our results contribute to showing that nasal chondrocytes may be less prone to fibrocartilage formation and less sensitive to inflammatory signals, confirming findings by other research groups [60].

In this model, however, the implants were placed parallel to the skin surface, an orientation that does not fully replicate the mechanical constraints encountered in human nasal reconstruction. This anatomical difference may affect local skin tension and, consequently, the mechanical environment acting on the implant, prompting us to test their stability in cadavers. First, casting the fibrin hydrogels around the full-scale silicone scaffolds dramatically improved the stiffness of the engineered constructs, approaching the rigidity and dissipation capacity of native nasal cartilage. The silicone scaffolds provided sufficient resistance to withstand surgical manipulation and the mechanical constraints of skin closure. The implant remained correctly positioned and structurally stable after implantation and nasal reassembly, without signs of deformation. This constitutes a preliminary assessment, and additional tests will be performed across multiple specimens with varying anatomies, to demonstrate the feasibility of our approach. Importantly, the silicone scaffold is 3D-printed, and its dimensions can be readily adapted upon fabrication. This will enable us to tailor the size and shape of the constructs to match the patient’s anatomy, in a personalized medicine approach. In addition, future work will now focus on pilot clinical trials to enable a comprehensive evaluation of long-term integration, mechanical resilience and functional performance in the dynamic and microbially active environment of the human nasal cavity.

Overall, these findings underscore the importance of combining biological and synthetic components to achieve both anatomical fidelity and mechanical robustness. This concept aligns with previous work in auricular cartilage engineering, where ear-shaped constructs, created using medical implants covered with engineered cartilage, retain their anatomical dimensions and mechanical strength after subcutaneous implantation in mice [61, 62]. In contrast, constructs lacking structural support frequently exhibited volume loss and deformation during *in vivo* maturation [63, 64]. Our study demonstrates that integrating a 3D-printed silicone scaffold effectively prevents deformation and collapse, which are issues that are frequently encountered in cartilage-only constructs.

## Conclusion

Collectively, this study establishes a proof of concept for a biologically active nasal implant capable of supporting cartilage regeneration, while meeting the mechanical demands of surgical manipulation. By combining a stable, 3D-printable silicone scaffold with a chondrocyte-laden fibrin hydrogel, and with human chondrocytes freshly extracted from the nasal septum, this hybrid strategy addresses key limitations of both purely synthetic and fully biological implants. While the current design is based on a standard L-strut geometry suitable for most clinical cases, the manufacturing process allows for customization based on the specific anatomy of the patient in an autologous approach. This would involve preoperative imaging, such as 3D nasal cavity scanning, enabling the fabrication of anatomically tailored constructs suitable with complex or atypical cases. Such flexibility opens avenues for truly personalized, anatomically integrated solutions in nasal reconstruction and potentially other cartilage repair applications.

## Supplementary Material

rbag122_Supplementary_Data
