# Supplementary material for: Bioengineered nasal septum implant with 3D-printed silicone and chondrocyte-seeded fibrin hydrogel
Source: Regen Biomater. 2026 Jun 9;13:rbag122. doi: 10.1093/rb/rbag122 (PMC13331287; doi:10.1093/rb/rbag122)
Supplement: rbag122_Supplementary_Data [file rbag122_supplementary_data.docx]

**Supporting Information**

**Supporting Information 1.** Microscopy images of 3D-printed silicone scaffolds. A) x4 and x10 magnification of scaffolds printed with a 40%, 60% or 70% porosity. Partial or complete pore closure was observed for porosities of 40% or 70%, but not for 60%. B) x4 and x10 magnification of scaffolds printed with 0.5 mm-wide or 1 mm-wide pores. Partial or complete pore closure was observed with a pore size of 0.5 mm, but not 1 mm.


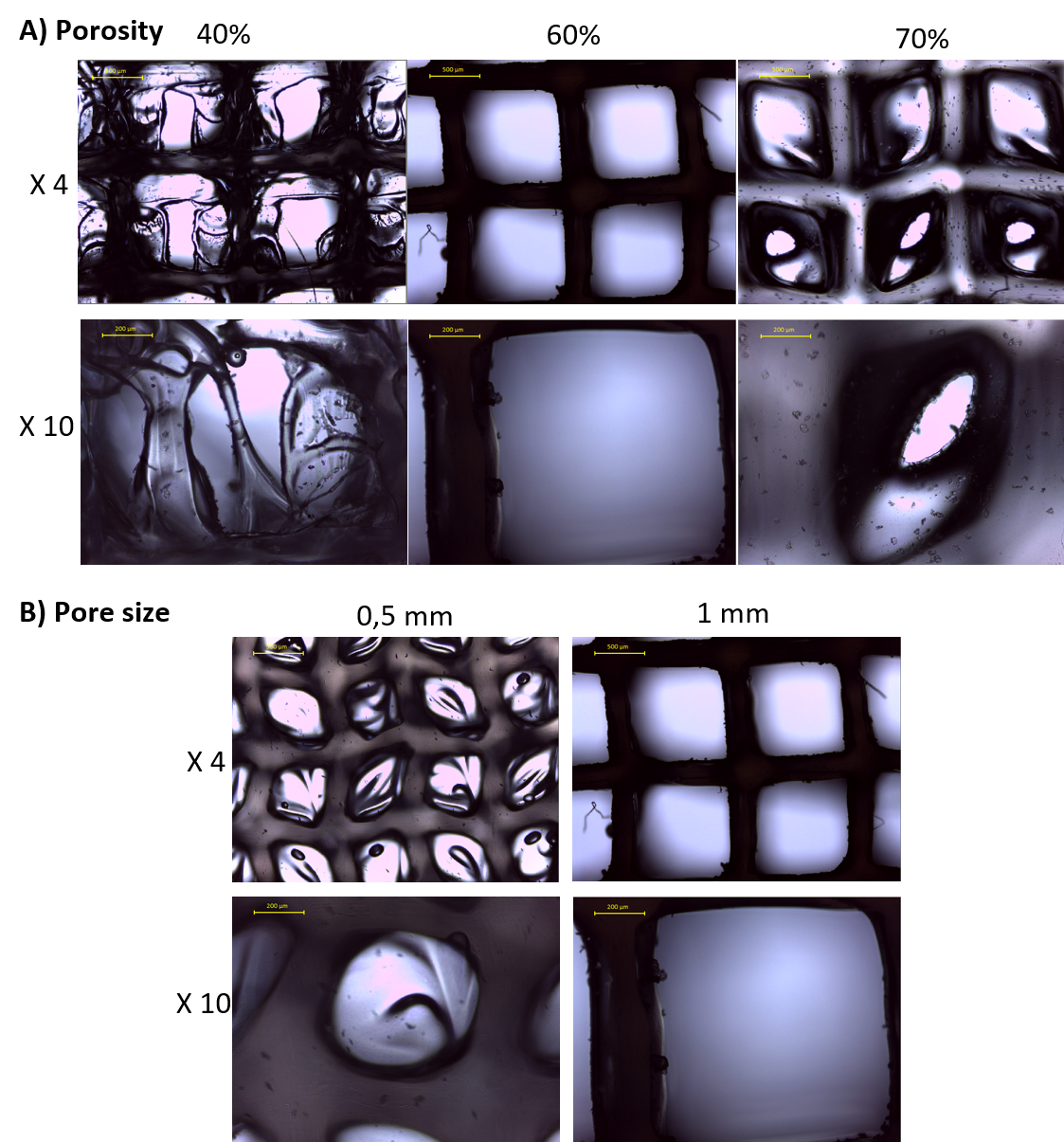


**Supporting Information 2.** A) Representative fields of view of live/dead assay performed on segments of fibrin/scaffolds construct cultured with nasal chondrocytes for 21 days in the presence of BIT. Black zone indicates silicone scaffold (scale bar = 1500 μm). Live cells appear in green, dead cells appear in red. B) Quantification of cell viability from three independent donors, after either 7 or 21 days of culture in fibrin hydrogels in the presence of the BIT cocktail. Statistical analysis was performed using Mann-Whitney’s test (N = 3). ns denotes not significant.


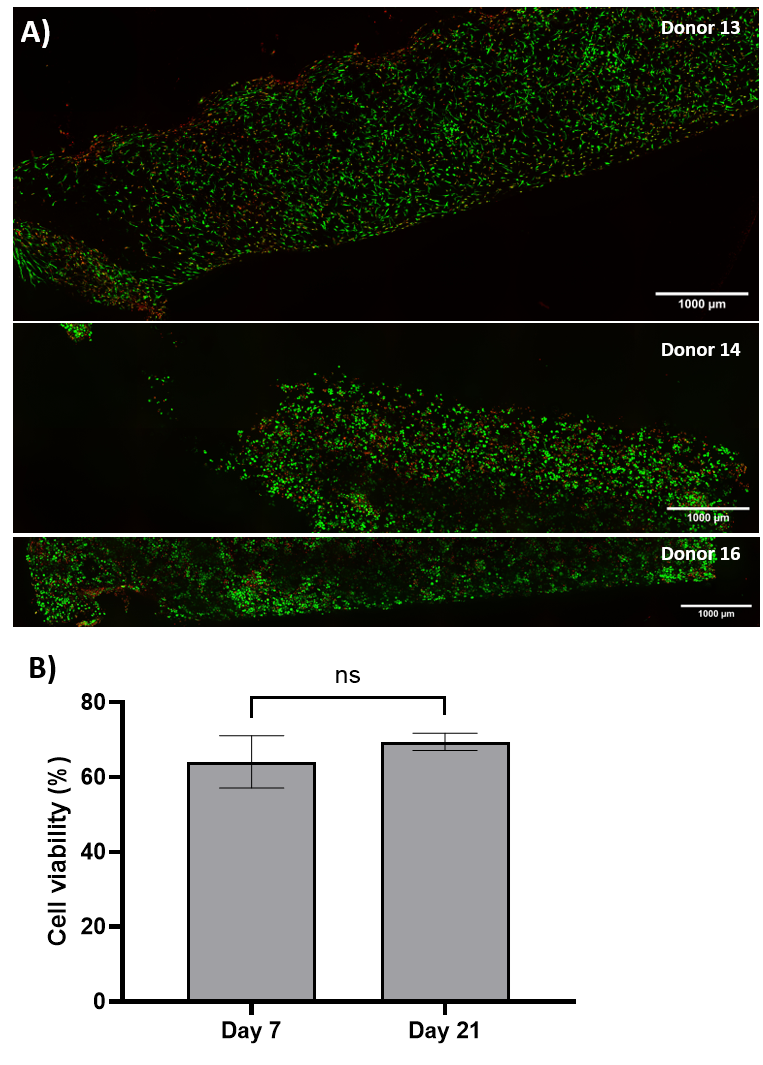


**Supporting Information 3.** Surgical feasibility test of the clinical model on human cadaver. (A) Removal of the native septal cartilage using an external rhinoseptoplasty approach. (B) Osteotomy at the nasal root to create mortise-and-tenon structure for stable implant positioning. (C) Insertion of the prototype between the mucosal layers using standard surgical instruments (Killian retractor and Adson forceps). (D) Comparison of the implanted construct (left) with a schematic basal view (right), showing anatomical congruence with the native septum. The nasal septum prosthesis reproduces the anatomical relationships of the native septum. (E) Initial patient profile before septal reconstruction. (F) Profile after implant placement, with skin repositioned to assess restoration of nasal morphology.


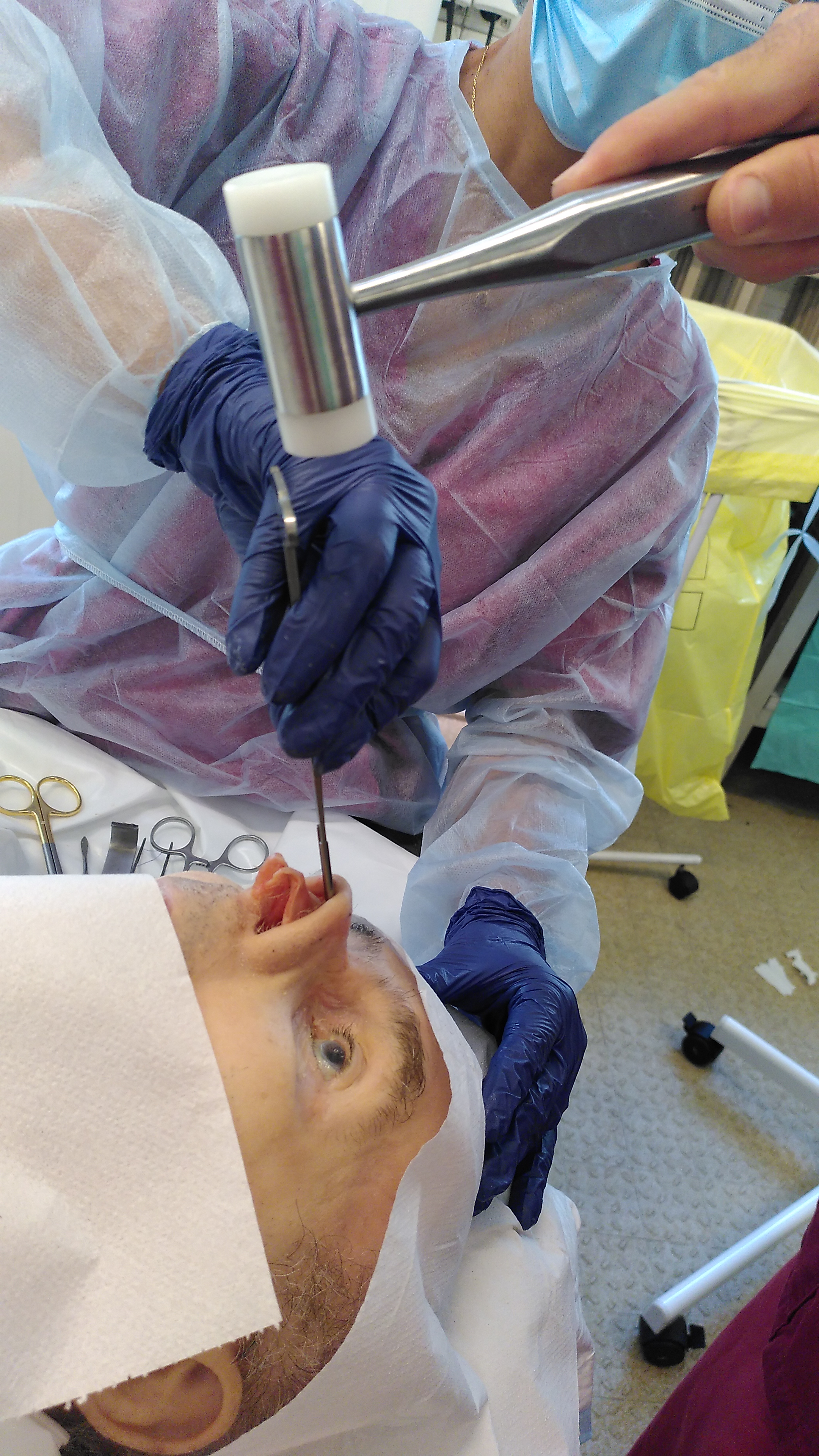

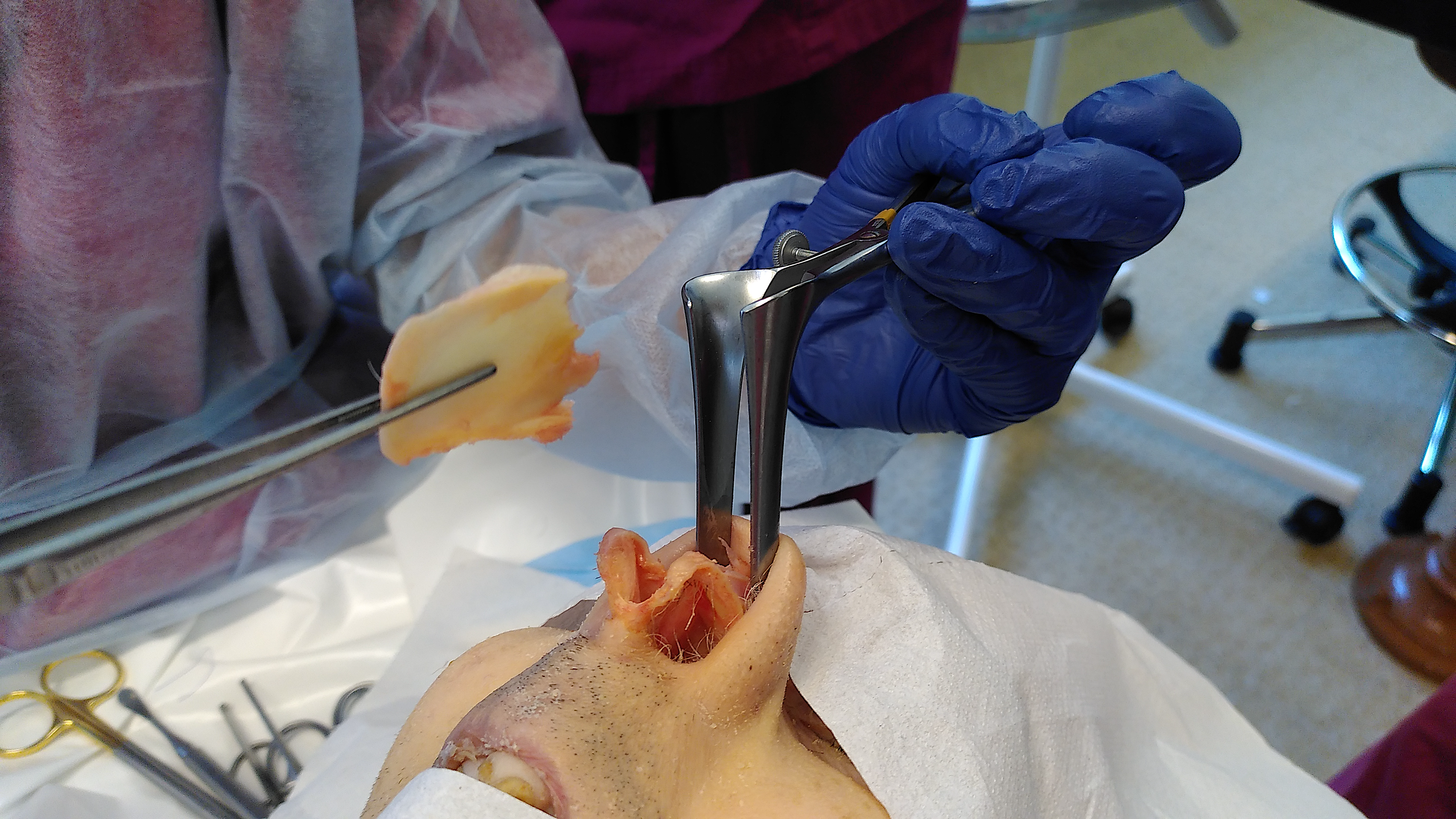

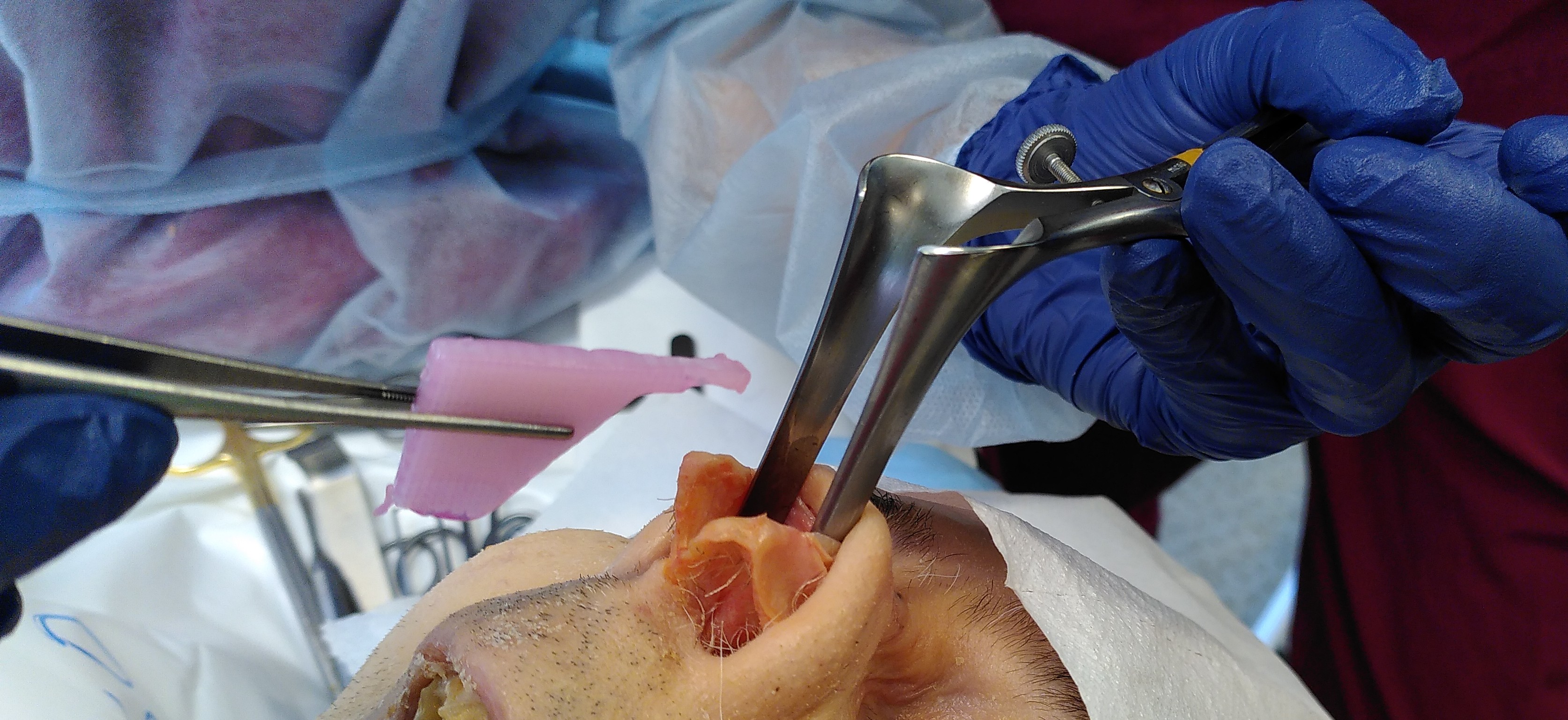


**(B)**

**(A)**

**(C)**


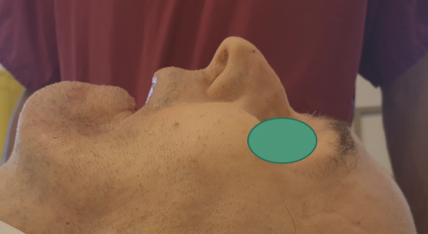

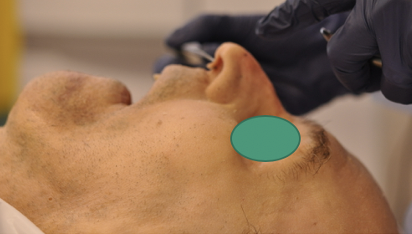


**(E)**

**(F)**


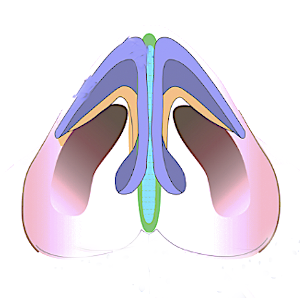

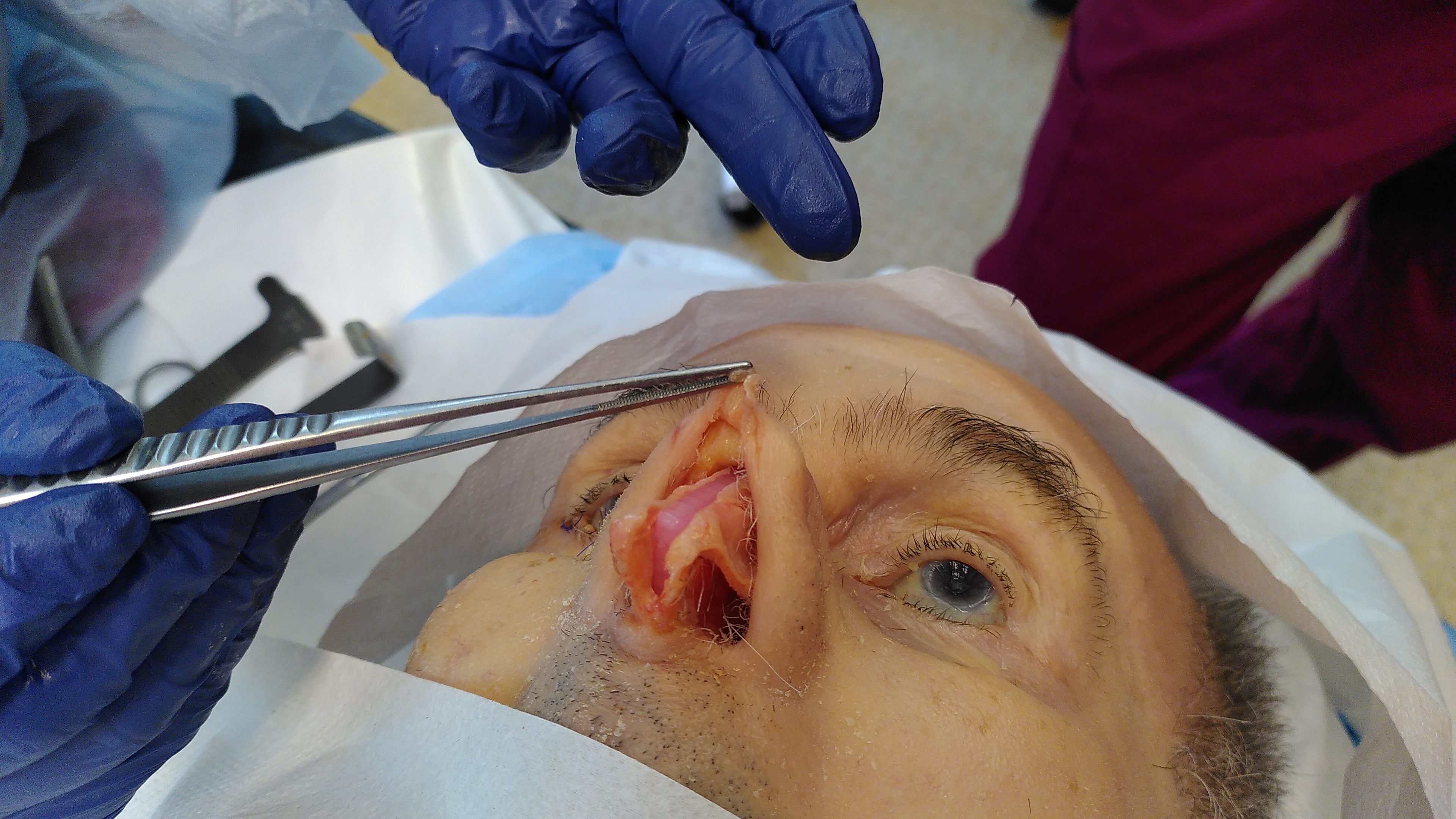


**(D)**

Alar cartilage

Engineered septum
